# Genome-Wide Identification and Expression Analysis of MTP (Metal Ion Transport Proteins) Genes in the Common Bean

**DOI:** 10.3390/plants12183218

**Published:** 2023-09-09

**Authors:** Hilal Yilmaz, Göksel Özer, Faheem Shehzad Baloch, Vahdettin Çiftçi, Yong Suk Chung, Hyeon-Jin Sun

**Affiliations:** 1 Plant and Animal Production Program, Izmit Vocational School, Kocaeli University, Kocaeli 41285, Türkiye; hilalayhanyilmaz@gmail.com; 2 Department of Field Crops, Faculty of Agriculture, Bolu Abant Izzet Baysal University, Bolu 14030, Türkiye; vahdet2565@yahoo.com; 3 Department of Plant Protection, Faculty of Agriculture, Bolu Abant Izzet Baysal University, Bolu 14030, Türkiye; gozer@gmail.com; 4 Faculty of Agricultural Sciences and Technologies, Sivas University of Science and Technology, Sivas 58140, Türkiye; 5 Department of Plant Resources and Environment, Jeju National University, Jeju 63243, Republic of Korea; yschung@jejunu.ac.kr; 6 Subtropical Horticulture Research Institute, Jeju National University, Jeju 63243, Republic of Korea; sunhj89@jejunu.ac.kr

**Keywords:** biofortification, Fe, Zn, MTP gene family, gene expression, hidden hunger

## Abstract

MTP/CDF carriers, called metal ion transport proteins, act as substrates for the transmission of micronutrients such as iron (Fe), zinc (Zn), and manganese (Mn) to membrane carriers in plants. In this study, genome-wide analysis of the MTP gene family in the common bean genome, expression analysis of the *PvMTP4*, *PvMTP5*, and *PvMTP12* genes after Fe and Zn treatments, and the effects of Fe and Zn applications on iron and zinc content were investigated. This study used common bean genotypes assumed to have high or low Fe and Zn accumulation ability. *PvMTP* genes were defined as containing conserved catalytic domains with molecular weights and protein lengths ranging from 41.35 to 91.05 kDa and from 369 to 813 amino acids (aa), respectively. As a result of the phylogenetic analysis, three main clusters containing seven subgroups were formed. In this study, the first characterization of the MTP gene family of beans was performed, and the responses of three different *PvMTP* genes in the Zn-CDF group to Fe and Zn applications were revealed. The obtained findings are thought to constitute pioneering resources for future research on common bean biofortification studies, plant breeding related to Fe and Zn, and the functional characterization of the MTP gene family.

## 1. Introduction

*Phaseolus vulgaris* L. (the common bean) is in the practical and economical crop group used to solve nutritional problems and eliminate protein deficits in the daily diet [1]. The common bean, one of the most widely grown edible legumes in the world, contains high amounts of protein (18–24%), carbohydrates (56%), and vitamins A, B, and C [2,3,4,5]. It is also rich in iron (Fe) and zinc (Zn) elements, which are defined as trace elements necessary for human health [6]. Commonly called the “Poor Man’s Meat” because of their high mineral, protein, and vitamin content, common beans are a vital food source for more than 300 million people worldwide [7]. According to the FAO, the world bean production area is 34.8 million ha, and the production amount was 27.55 million t in 2020 [8]. It is estimated that 9.5 billion people will live in the world by 2050, and part of the population will be exposed to hunger due to climate changes that adversely affect agriculture [5,9]. Today, it is known that more than 800 million people worldwide are undernourished [10]. Decreased access to food leads to malnutrition in terms of vitamins, minerals, and hidden hunger. Hidden hunger, caused by an energy-dense but nutrient-poor diet, is due to various micronutrient deficiencies, primarily iron, zinc, iodine, and vitamin A [11]. Approximately 1.2 billion individuals worldwide suffer from iron deficiency anemia [12], and 2 billion suffer from zinc deficiency [13].

In recent years, biofortification has been used as an effective plant breeding method to address hidden hunger and ameliorate human micronutrient deficiencies [14,15,16]. The technique is based on improving the nutritional quality of the edible parts of plants through conventional or transgenic approaches [17]. Identifying the gene families involved in biological processes such as the uptake, accumulation, and distribution of metal ions in plants will accelerate biofortification breeding studies [18]. Metal ion transport proteins (MTP) are one of the critical gene families involved in the flow of ions to subcellular organelles or out of the cytoplasm within the cell [19]. MTP gene members, called cation diffusion facilitators (CDF), are responsible for the homeostasis of many metal ions, such as Mn, Fe, and Zn [20,21]. 12 MTP genes have been described in *Arabidopsis thaliana*, but their functional characterizations have yet to be discovered [22]. In Arabidopsis, AtMTP1 has been involved in Zn tolerance [23]. AtMTP3 can increase plant tolerance to Zn and Co, and Fe deficiency increases Zn accumulation in Arabidopsis leaves [24]. The AtMTP11 gene can be usable in defining Mn sensitivity [25]. So far, the functional properties of MTPs in *Oryza sativa* (*OsMTP*) [20,21,26], *Triticum aestivum* (*TaMTP*) [27], *A. thaliana* (*AtMTP*) [24,28,29], *Medicago truncatula* (*MtMTP*) [30], *Glycine max* (*GmMTP*) [31], and *Nicotiana tabacum* (*NtMTP*) [32] have been characterized. 

The genetic mechanism of the response of MTP genes to iron (Fe) and zinc (Zn) elements in the bean plant has yet to be clarified. This study identified members of the MTP gene family in the common bean genome. In addition, it aimed to determine the expression levels of *PvMTP* (4,5,12) genes and evaluate Fe and Zn accumulation in common bean pods and roots.

## 2. Results and Discussion

### 2.1. Identification of MTP Genes in the Common Bean

**Figure 1:**
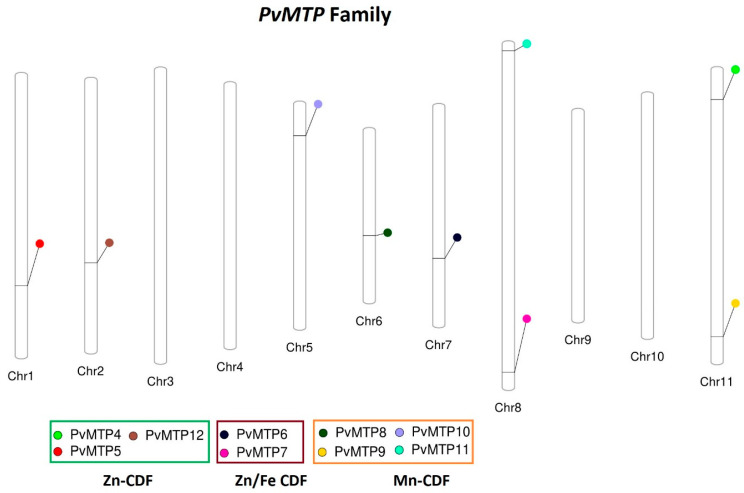
Locations of *PvMTP* genes in common bean chromosomes.

A total of nine *PvMTP* (*PvMTP4*, *PvMTP5*, *PvMTP6*, *PvMTP7*, *PvMTP8*, *PvMTP9*, *PvMTP10*, *PvMTP11*, and *PvMTP12*) *P. vulgaris* genes with high similarity to the protein sequences of *A. thaliana* were discovered via bioinformatic analysis. All the *PvMTP* genes were found in seven of eleven common bean chromosomes (Chr). The *PvMTP4* and *PvMTP9* genes were located on Chr 11, the *PvMTP5* gene on Chr 1, the *PvMTP6* gene on Chr 7, the *PvMTP7* and *PvMTP11* genes on Chr 8, the *PvMTP8* gene on Chr 6, and the *PvMTP12* gene on Chr 2 (Figure 1). The identified *PvMTP* genes were revealed to have encoded proteins with 369–813 aa; their molecular weights ranged from 41.35 to 91.05 kDa, and their protein isoelectric points were between 5.08 and 8.97 (Table 1, Figure 1).

### 2.2. Phylogenetic Analysis of PvMTP Gene Families in the Common Bean

**Figure 2:**
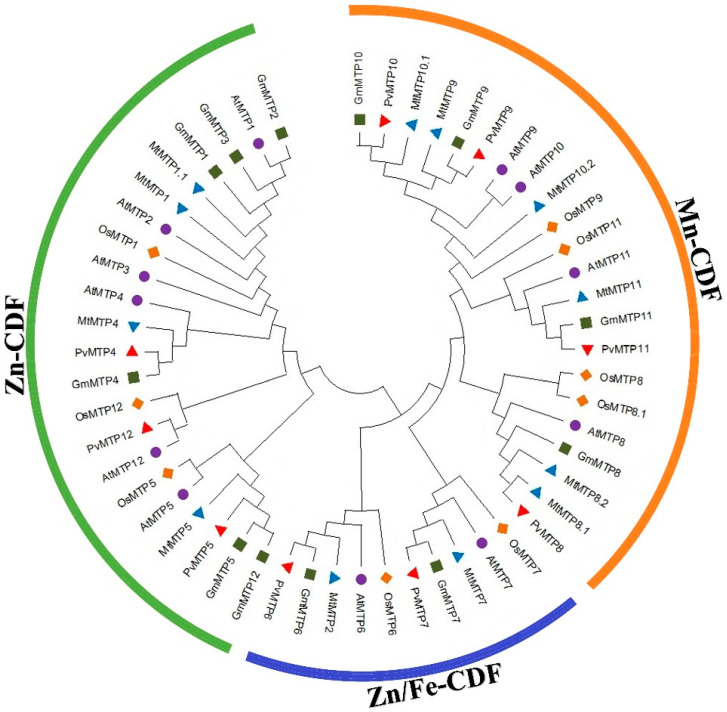
Phylogenetic analysis of Phaseolus vulgaris (Pv), Arabidopsis thaliana (At), Medicago truncatula (Mt), Oryza sativa (Os), and Glycine max (Gm).

Phylogenetic analysis was performed using the MTP protein sequences of different plants, such as the common bean (*PvMTP*s), Arabidopsis (*AtMTP*s), the soybean (*GmMTP*s), Medicago (*MtMTP*s), and rice (*OsMTP*s). Three main clusters emerged in the phylogenetic tree. *PvMTP4*, *PvMTP5*, and *PvMTP12* genes were found in cluster I, *PvMTP6* and *PvMTP7* genes in cluster II, and *PvMTP7*, *PvMTP8*, *PvMTP9*, *PvMTP10*, and *PvMTP11* genes in cluster III (Figure 2). The distribution in the phylogenetic tree appears to be consistent with previously defined MTP groups [19,20,23,33,34,35].

According to Gustin et al. [36], MTP sequences are divided into seven subgroups (1, 5, 6, 7, 8, 9, and 12). *MTP1*, *MTP2*, *MTP3*, and *MTP4* are in group 1, *MTP5* is in group 5, *MTP6* is in group 6, *MTP7* is in group 7, *MTP8* is in group 8, *MTP9*, *MTP10*, and *MTP11* are in group 9, and *MTP12* is in group 12. It is assumed that 1, 5, and 12 groups constitute Zn-CDF, 6 and 7 groups are Fe/Zn-CDF, 8 and 9 groups are Mn-CDF [27,33]. All proteins formed the same group except for *MtMTP2*, which was also determined to be in group 6 in previous studies [34]. The fact that most *PvMTP*s belong to the group showing specificity for Mn and Zn suggests that these genes may play important roles in Mn and Zn homeostasis in common beans.

### 2.3. Motifs and Exon/Intron Organization

**Figure 3:**
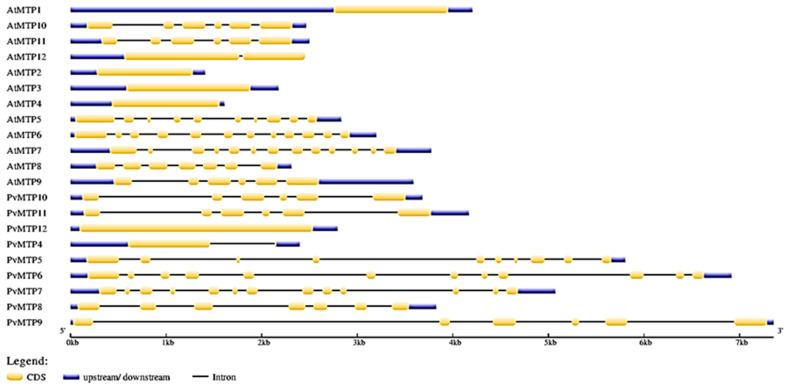
MTP gene exon/intron analysis in Arabidopsis (At) and the common bean (Pv).

**Figure 4:**
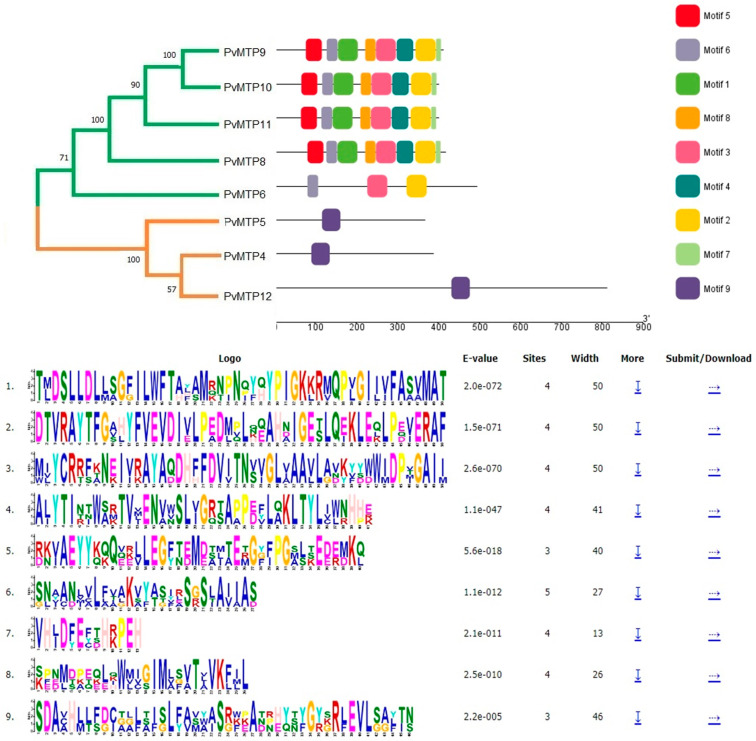
Conserved motifs of *PvMTP* proteins.

The common bean and Arabidopsis MTP sequences were compared, and the exon-intron structure was determined via mapping genome sequences (Figure 3). The nine conserved motifs of the metal tolerance protein sequence were obtained using the MEME SUITE tool (Table 2). Similar motif compositions seemed to have the same phylogenetic group in MTP members (Figure 4).

The exon and intron regions of the MTP protein sequences of *A. thaliana* and *P. vulgaris* plants were analyzed, and it was found that *AtMTP9*, *AtMTP10*, *AtMTP11*, *PvMTP9*, *PvMTP10*, and *PvMTP11* consisted of six exons and five introns. Also, *AtMTP8* and *PvMTP8* had seven exons and six introns; *AtMTP6*, *AtMTP7*, *PvMTP6*, and *PvMTP7* had thirteen exons and twelve introns; *AtMTP5* and *PvMTP5* had ten exons and nine introns; *AtMTP1*, *AtMTP2*, *AtMTP3*, and *AtMTP4* had no intron regions. *PvMTP4* had two exons and one intron, while *PvMTP12* did not contain an intron region (Figure 3). The distribution of the exon and intron numbers of the MTP gene family, divided into three main groups—Zn-CDF, Zn/Fe CDF, and Mn-CDF in the phylogenetic tree—had similar results. For group 1, also known as Zn-CDF, it was determined that most members contained no intron, while the *PvMTP4* and *AtMTP12* members had one intron. The highest number of introns was found in group 2, Zn/Fe CDF, (11–12 introns), while all members of group 3, Mn-CDF, had five introns. The conserved motif sequences that were obtained were identified in the UniProt and Pfam online databases (Figure 4). Motif 1, Motif 2, Motif 3, and Motif 9 may be cation efflux proteins, while Motif 4 and Motif 8 may be ZT-Dimer zinc carrier dimerization proteins. The other conserved motifs (Motifs 5, 6, and 7) could not be defined using the UniProt and Pfam databases.

**Figure 5:**
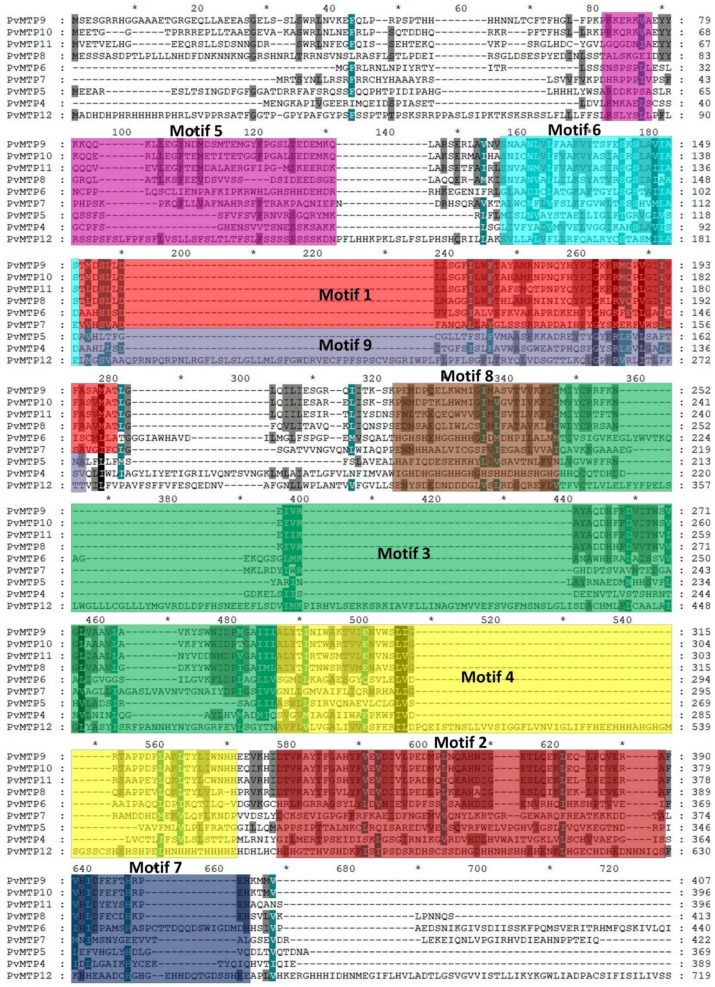
Multiple sequence alignment of *PvMTP* proteins using ClustalW. Dark or light shading designates similar amino acids.

In a study of wheat (*T. aestivum*) metal tolerance proteins, five motifs were identified; one of these motifs was reported to be a cation efflux protein, while the other one was a ZT-Dimer (PF16916) protein, and the remaining three motifs were not associated with any location [27]. The results of motif analysis were evaluated using the cluster formed by the *PvMTP* sequences. It was observed that the motifs were divided into three main groups: Zn-CDF, Zn/Fe CDF, and Mn-CDF. There was one motif in the Zn-CDF group, there were three motifs in the Zn/Fe CDF group, and there were eight motifs in the Mn-CDF group. It was predicted that the functional roles of MTP genes with the same motifs may also be highly similar [27]. The groups in the cluster analysis of *PvMTP*s were supported by the unique motif analysis of each of their genes. A motif was not detected only in the *PvMTP*7 protein sequence in this study. Studies on the identification of MTP genes in soybean and rice have shown that no motif belonging to the *MTP7* gene was identified [22,35]. The identified *PvMTP* sequences were multiple-aligned using ClustalW and shaded using GeneDoc with similar residues in gray and black (Figure 5).

The shaded sequences revealed that the sequences of the Mn-CDF members were more conserved than the others. In a study conducted with *Citrus sinensis* MTPs, *CitMTP*s of the Mn-*MTP* subgroup were found to be more highly conserved than those of the other two subgroups [36]. The findings of the study conducted on wheat also suggested that the sequences of the Mn-*MTP* members are more conserved [27].

### 2.4. Promoter Region Cis Motif Analysis of PvMTP Genes

Analysis of promoter cis-elements is an important method used to understand gene function [37]. Learning about gene domains upstream of the transcription start site is essential to understand the mechanism of genes at the transcriptional level [38]. According to cis-regulatory element analysis, a total of 38 different cis-regulatory element motifs with the potential to regulate gene expression in response to three groups (cis-acting elements, stress and growth elements, and hormone-sensitive elements) were identified in *PvMTP* genes (Table 3). The frequency in the regulatory region of each gene corresponding to these elements and their overall frequency in family members vary widely [27]. The most common elements in all *PvMTP* genes are CAAT and TATA motifs. In studies on *T. aestivum*, *G. max*, *M. truncatula*, *Lotus japonicus*, *N. tabacum*, and *Vitis vinifera* MTP genes, CAAT and TATA motifs were observed to be the most common motifs [27,37,39,40]. The most common cis-regulatory element after CAAT and TATA motifs in *PvMTP* genes was associated with light responsiveness elements, such as G-box, GT1-motif, the 3-AF1 binding site, TCCC-motif, GATA-motif, Sp1, AE-box, I-box, LAMP-element, MRE, TCT-motif, chs-CMA2a, Box 4, ATCT-motif, chs-CMA1a, ATC-motif, AT1-motif, LTR, MBS, O2-site, circadian, ARE, CAT-box, and TC-rich repeats (Table 3). In other MTP identification studies, the most common motifs after the cis-acting factor group have been light responsiveness elements [27,37,39,40]. Additionally, abiotic stress elements comprised LTR for low-temperature responsiveness, MBS for drought inducibility, the O2- site for zein metabolism regulation, circadian elements for circadian control, ARE for anaerobic induction, CAT-box for meristem expression, and TC-rich repeats for defense and stress responsiveness. Also, motifs carrying hormonal responses, such as auxin responsiveness, methyl jasmonate (MeJA) responsiveness, abscisic acid responsiveness, salicylic acid responsiveness, and gibberellin responsiveness, have been associated with *PvMTP* genes. Considering the presence of all motif elements, it was concluded that *PvMTP* genes can be transcriptionally controlled using various stimuli and can participate in different metabolic processes in plants [40].

### 2.5. Protein–Protein Interaction Analysis among the PvMTP Family Members

**Figure 6:**
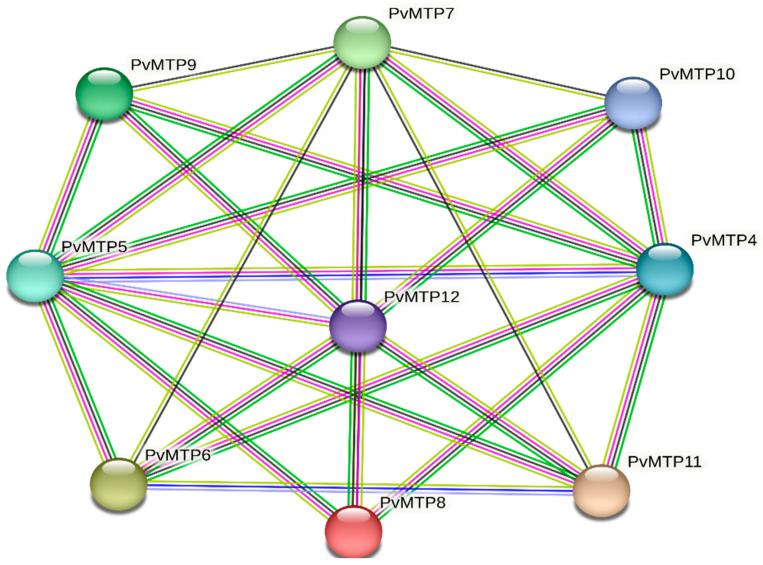
Protein—protein interaction between *PvMTP* family members. The type of interaction evidence is indicated by the line color (green: gene neighborhood; light blue: curated databases; pink: experimentally determined; dark blue: gene co-occurrence; yellow: text-mining; black: co-expression).

Protein–protein interactions were obtained from the String (https://string-db.org/ accessed on 2 December 2022) online database. The results showed protein–protein interactions within *PvMTP* proteins where the total number of nodes was nine with an average of 5.78 and edges of 26 (Figure 6). Furthermore, the analysis showed PF16916 (five proteins) and PF01545 (nine proteins) domains within the *PvMTP* protein family. These domains confirmed our previous analyses. The PF16916 domain is defined as the dimerization domain of zinc transporters and the PF01545 domain as the cation efflux family. In a study of tomato MTP proteins, 11 node numbers with an average of 7.09 and 39 edges were observed. Also, it was reported that the protein family contains PF16916 (seven proteins) and PF01545 (ten proteins) domains [34]. Protein–protein interaction analysis provides information about the interactions between the environment and plant development processes [41]. String gene ontology results revealed that *PvMTP* genes show several different molecular functions, such as zinc homeostasis, zinc active transmembrane transport, iron ion homeostasis, active cadmium transmembrane transport, and active manganese ion transmembrane transport. This suggests that *PvMTP* genes are included in the cellular cadmium, zinc, manganese, and iron ion homeostasis biological processes. Also, these genes were found to be part of different cellular components, such as the vacuolar and plasma membranes. *GmMTP* genes are known to be included in biological processes, such as cellular zinc, cadmium, and iron ion homeostasis in soybeans [22]. The soybean study supported the results of *PvMTP* protein–protein interaction analysis.

### 2.6. Subcellular Localizations and Gene Ontology of the PvMTP Gene Family

**Figure 7:**
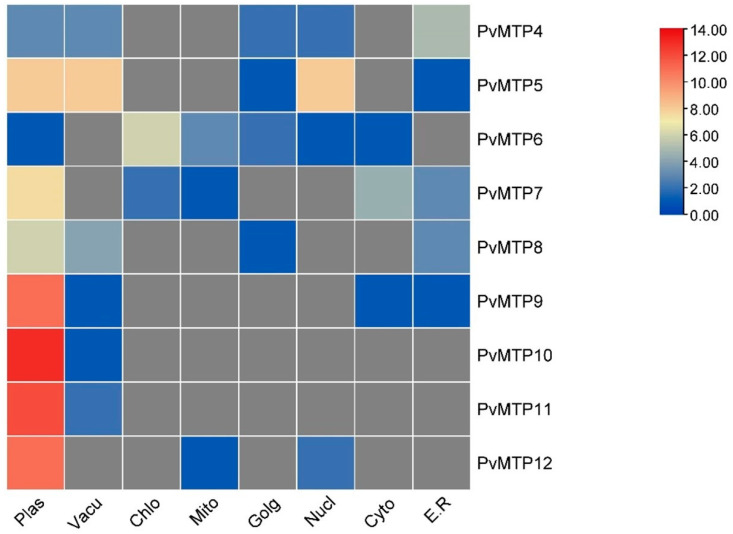
Heatmap of subcellular localization of *PvMTP* genes (plas: plasmid; vacu: vacuole; chlo: chloroplast; mito: mitochondria; golg: Golgi apparatus; nucl: nucleus; cyto: cytoplasm; e.r.: endoplasmic reticulum).

Through using different online platforms such as WoLF PSORT and CELLO, predictions can be made about the subcellular locations of genes. For the subcellular localization of *PvMTP* genes, it was determined in the Cello database that seven *PvMTP*s are located on the plasma membrane, and *PvMTP6* and *PvMTP7* are in the mitochondria. Detailed search results are given in Figure 7. When the subcellular localization analysis results of the MTP gene family were examined, it was observed that most genes were localized in the tonoplast (vacuolar membrane), while some were localized in the cytoplasm or chloroplast [27,34]. In a study of *SlMTP* genes (*Solanum lycopersicum*), it was determined that *SlMTP*1,2,3,3,5,5,7,9,10 was located in the vacuole membrane (tonoplast), *SlMTP*5,8,11 in the cytoplasm, and *SlMTP*6 in the chloroplast [34]. Most of the 10 *VtMTP (V. vinifera)* genes were found to be localized in the vacuole, except for *VtMTP*9.1 [39]. *MtMTP* genes (*M. truncaluta*) were observed to be in the cell membrane, plasma membrane, vacuole, Golgi apparatus, and root hairs [37].

**Figure 8:**
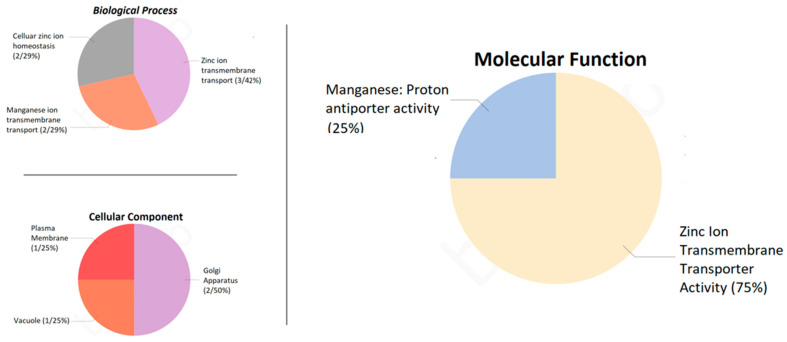
Gene ontology analysis of *PvMTP* gene family.

Functional analysis of the protein sequences of the *PvMTP* genes was performed using Blast2GO 5.2.5 (https://www.blast2go.com/ accessed on 3 December 2022), and the biological processes, molecular processes, and cellular components of nine MTP proteins were determined [42,43]. The biological processes of the *PvMTP* proteins were divided into three different groups: cellular zinc homeostasis (29%), zinc transfer (43%), and manganese transfer (29%). The molecular functions of the metal tolerance proteins were determined as 25% manganese activity and 75% zinc transmembrane transport activity. The cellular components were found to be 25% in the plasma membrane, 25% in the vacuole, and 50% in the Golgi apparatus (Figure 8).

### 2.7. Effect of Zn and Fe Application on PvMTP4, PvMTP5, and PvMTP12 Genes in Common Bean Genotypes

The expression levels of the *PvMTP* genes in pod tissues (3, 6, 12, and 24 days) of Fe- and Zn-treated bean genotypes were determined using qRT-PCR analysis (Figure S1). Fe (0, 15, and 30 µM) was applied to the EL-29 (Fe low storage) and MŞ-46 (Fe high storage) genotypes, and Zn (0, 10, and 15 µM) was applied to the Bn-16 (Zn low storage) and Sv-12 (Zn high storage) genotypes. Most of the Fe and Zn treatment results indicated that the *PvMTP4*, *PvMTP5*, and *PvMTP12* genes were up-expressed in the pod tissues (3, 6, 12, and 24 days) of four cultivars. The *PvMTP4* gene expression levels significantly increased via the Fe and Zn treatments in all genotypes, except for the 3-day pod of the EL-29 genotype. The highest gene expression level was achieved via the 30 µM of Fe and 15 µM of Zn applications. This increase was remarkable in the zinc-treated BN-16 and SV-12 genotypes. The highest gene expression level for the 30 µM of Fe application was detected in the 24-day pods (2.60-fold) of the EL-29 genotype. This increase was 2.24-fold in the 6-day pods of the MŞ-46 genotype. The highest gene expression levels of the BN-16 and SV-12 genotypes were determined in the 6-day pods (5.98- and 6.32-fold) with the 15 µM of Zn application. The highest gene expression levels of the *PvMTP4* gene were observed in the Zn application. When the *PvMTP5* gene was examined, it was observed that the expression level showed a significant increase in all genotypes. This increase was greater in the BN-16 and SV-12 genotypes treated with zinc compared to the iron-treated genotypes. The *PvMTP5* gene expression level in the 6-day pod of the EL-29 genotype treated with 30 µM of Fe showed a 4.99-fold increase compared to the housekeeping gene. This increase was 2.95-fold in the 3-day pod of the MS-46 genotype treated with 30 µM of Fe. The highest gene expression levels in the BN-16 and SV-12 genotypes treated with 15 µM of Zn were determined in the 6-day pods (15.22- and 11.62-fold). In all genotypes, the *PvMTP12* gene expression levels significantly increased via the 30 µM of Fe and 15 µM of Zn treatments. This increase was higher in the BN-16 and SV-12 genotypes treated with Zn than those treated with Fe. The highest gene expression increase was realized in the 12-day pods of the EL-29 genotype (8.30-fold) and 6-day pods of the MŞ-46 genotype (5.07-fold). For the BN-16 and SV-12 genotypes treated with zinc, the highest gene expression levels were determined in 6-day pods (118.24- and 69.57-fold). The greatest change was observed in the *PvMTP12* gene with the 15 µM of Zn treatment. When the differences between the Fe and Zn treatments were analyzed, it was determined that the *PvMTP4*,*5*,*12* gene was more effective in the SV-12 genotype with a high zinc storage ability. 

**Figure 9:**
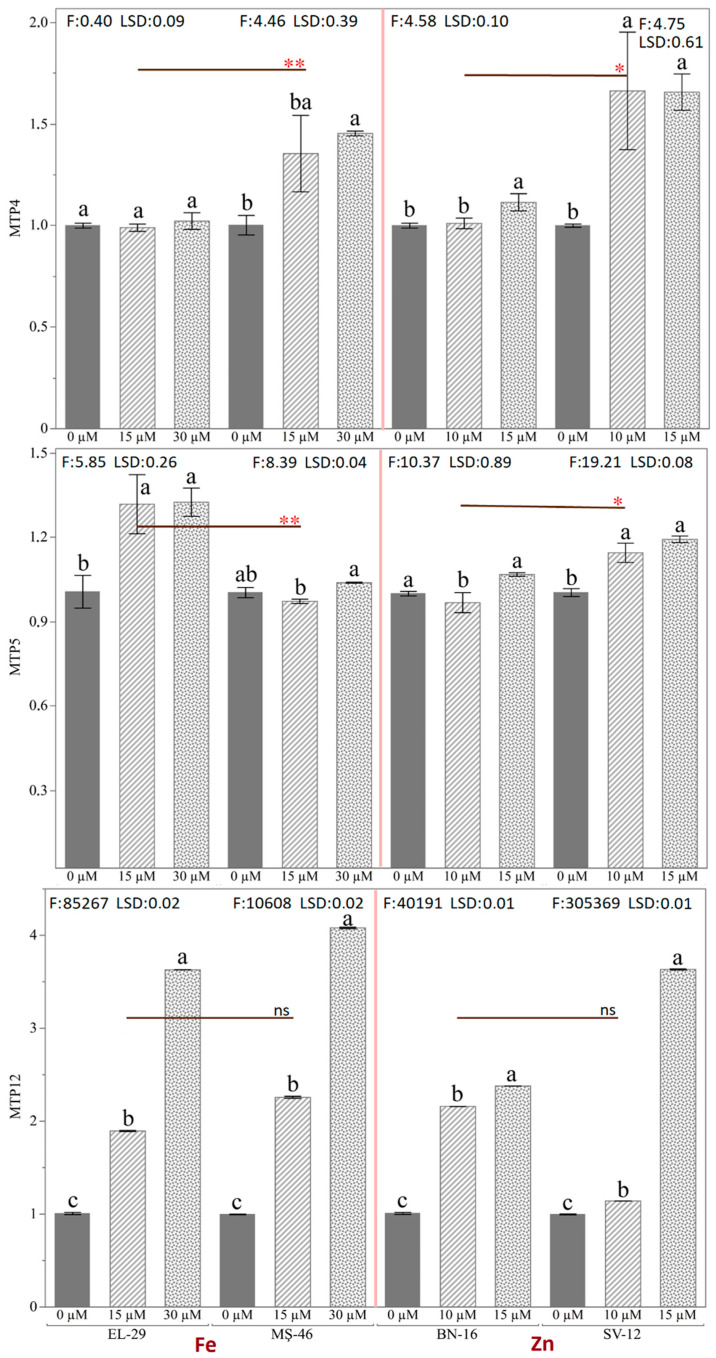
Expression levels of *PvMTP* (4, 5, 12) genes in the root of common bean genotypes after Fe (0, 15, and 30 µM) and Zn (0, 10, 15 µM) applications (Different letters indicate significant differences according to Student’s t-test, * (*p* ≤ 0.05); ** (*p* ≤ 0.01); ns: non-significant).

The expression levels of the *PvMTP12* gene excessively increased compared to the housekeeping gene in the root tissue of four bean genotypes with different iron and zinc storage abilities (Figure 9). The most significant increase in gene expression was realized via the application of 30 µM of Fe and 15 µM of Zn. For the *PvMTP4* gene, the application difference was significant in the three genotypes other than the EL-29 genotype. The gene expression level of the MŞ-46 genotype treated with 30 µM of Fe increased 1.04-fold, and in the other genotypes (MŞ-46, SV-12) treated with 15 µM of Zn, the gene expression levels increased 1.01- and 1.65-fold, respectively. These increases in the *PvMTP5* gene were 1.32-, 1.03-, 1.07-, and 1.19-fold in genotypes EL-29, MŞ-46, BN-16, and SV-12, respectively. Although the statistical difference between the applications was significant, the fold changes of the two genes were small in the root tissue. In the *PvMTP12* gene, the increase in gene expression levels was remarkable for all treatments compared to the control. The increases in the EL-29 and MS-46 genotypes via the 30 µM of Fe application were 3.63- and 4.08-fold, respectively. The change in the gene expression levels of the BN-16 and SV-12 genotypes, to which 15 µM of Zn were applied, were 2.37- and 3.63-fold, respectively. Studies on Arabidopsis plants were examined, and it was found that *AtMTP1* is expressed in all plant tissues and is capable of transporting Zn to the vacuoles [23], while the *AtMTP3* and *AtMTP8* genes contribute to metal tolerance by transporting Zn and Mn, respectively [24,44]. Also, *AtMTP11* was found to increase Mn tolerance in plants by transferring Mn to the endosomal vesicles [25,45]. The response of the *GmMTP* genes in the case of iron toxicity and deficiency in soybeans was investigated. The *GmMTP4* and *GmMTP12* genes did not respond, while the gene expression levels of the *GmMTP5* gene decreased compared to the control [22]. Medicago truncatula was assessed for the responses of different elements, such as Cd, Co, Fe, Mn, and Zn, on the *MtMTP* genes in another study. It was found that the Fe and Zn treatments caused a decrease in *MtMTP4* gene expression, that the *MtMTP5* gene was more expressed in both leaves and roots via the Zn treatment compared to Fe, and *MtMTP12* gene expression increased via the Fe and Zn treatments [34]. The expression level of the *NtMTP4* and *NtMTP12* genes decreased via the Fe and Zn applications compared to the control, and *NtMTP5* was more expressed in the root [43]. The responses of different elements, such as Cd, Co, Fe, Mn, and Zn, on the *SlMTP* genes of tomato plants were examined, and the *SlMTP4* and *SlMTP5* genes were reported to be highly expressed in both root and leaf Fe applications [34]. In this study, although the expression levels of the *PvMTP4*, *PvMTP5*, and *PvMTP12* genes in the pods of EL-29 and MS-46 genotypes treated with Fe increased compared to the control, the highest expression level was determined in the BN-16 and SV-12 genotypes treated with Zn. Variation was also high between the BN-16 and SV-12 genotypes with different zinc storage abilities. In the root, the highest gene expression was in the *PvMTP12* gene. When the results were evaluated together, it was seen that MTP genes may differ during the various growth and development stages of different plants.

### 2.8. Effect of Fe and Zn Application on Fe and Zn Accumulation in Common Bean Genotypes

Common bean pods (at 3, 6, 12, and 24 days) and root tissues with Fe and Zn contents were determined via atomic absorption spectroscopy analysis. Iron content was analyzed in the pods of the EL-29 genotypes with a low Fe storage ability and MŞ-46 with a high iron storage ability. Except for the 24-day pods, the pods of the MŞ-46 genotype contained high iron content on all days. The highest iron content was determined as 257 mg/kg in the 3-day pods of the MŞ-46 genotype. The Zn content of Fe-treated EL-29 and MŞ-46 genotypes decreased with increasing iron doses. While 63.8 mg/kg of zinc content was determined for the control treatment, 38.57 mg/kg of zinc content was determined for the 30 µM of Fe treatment in the 3-day pods of the MŞ-46 genotype. A decrease of approximately 39% was observed compared to the control. When the Fe content In the pods of the zinc-treated BN-16 and SV-12 genotypes was analyzed, the responses of the genotypes to the Zn application were different. In the BN-16 genotype, which was assumed to have a low zinc storage ability, the Zn application increased the iron content compared to the control. While the control contained 75.3 mg/kg of Fe in the 24-day pods, the 15 µM of Zn treatment realized 143.9 mg/kg of Fe. The application increased the iron content in the pods by approximately 91%. In the SV-12 genotype, which was assumed to have a high zinc storage ability, the Zn treatment decreased the iron content in the 6-, 12-, and 24-day pods. While the control contained 129 mg/kg of Fe in the 12-day pods, the 15 µM of Zn treatment achieved 91 mg/kg of Fe. The application reduced the iron content in the pods by about 30% (Figure S2). The iron application decreased the zinc content in the pods of the EL-29 and MŞ-46 genotypes. 

According to the results of the Fe analysis of the root tissue, the highest accumulation was observed in the MŞ-46 genotype (1814 mg/kg) to which 30 µM of Fe was applied. The zinc application increased the iron accumulation in the root. The Fe application decreased the Zn accumulation in the root. This decrease was higher in the MŞ-46 genotype, which was thought to have a high Fe storage ability. The highest zinc accumulation in the root tissue was determined in the SV-12 genotype (265.8 mg/kg). It is known that iron–zinc interaction exists in many plants. In the plant root zone, there is competition for absorption between Fe^+3^ and Zn^+2^ ions [46]. The inhibitory effect of iron on zinc uptake reduces the transport and accumulation of zinc with increasing iron levels. As a result, the zinc uptake via the root decreases [47]. In studies of Arabidopsis, Fe deficiency has resulted in Zn accumulation, while Zn excess has resulted in physiological Fe deficiency [48,49]. Researchers have reported that iron varies between 40.0 and 182.45 ppm and that zinc varies between 17.7 and 66.68 ppm in the seed mineral concentrations of bean genotypes [50,51]. There is high variation in Fe and Zn among common bean varieties [52,53]. The most important reason for this is that there are genotypes with higher Fe concentrations in the Andean genome pool [54], while there are genotypes with higher zinc content in the Meso-American gene pool [55].

## 3. Materials and Methods

### 3.1. Plant Material

The seeds of four common bean varieties named BN-16 (low Zn accumulation), SV-12 (high Zn accumulation), EL-29 (low Fe accumulation), and MŞ-46 (high Fe accumulation) were used in this study. The Fe and Zn accumulation performance of these genotypes was determined via TÜBİTAK project No. 215O630 (Identification of Important Agronomic, Physicochemical, Mineral, Antioxidant and Cooking Properties of Bean Gene Resources in Turkey by Genome Diameter Association Studies (GWAS)).

### 3.2. Identification of MTP Genes in Common Beans

The conserved domains of the MTP genes in the common bean were obtained from Phytozome database 13 (www.phytozome.net/ accessed on 3 December 2022). The sequence data and gene ID numbers of genes with high similarity (>70%) were obtained via BLASTn analysis. The nucleotide sequences of the MTP genes belonging to *A. thaliana* (At), *G. max* (Gm), *O. sativa* (Os), and *M. truncatula* (Mt) were determined online using NCBI (https://www.ncbi.nlm.nih.gov/ accessed on 3 December 2022) and Uniprot (https://www.uniprot.org/ accessed on 3 December 2022). Gene identifier information was searched for using “IDs” as a keyword on the NCBI (http://www.ncbi.nlm.nih.gov/ accessed on 3 December 2022) platform, and the chromosomes on which the genes were located were determined. The PFAM database (https://pfam.xfam.org/ accessed on 3 December 2022) was used to validate the protein domains of the MTP sequences. The physicochemical properties of the identified proteins, such as their molecular weights, theoretical isoelectric points (pI), and instability indices, were detected via the online database ProtParam Tool (http://web.expasy.org/protparam/ accessed on 3 December 2022).

### 3.3. Chromosome Localization and Phylogenetic Analysis

The protein sequences of *A. thaliana* (At), *O. sativa* (Os), *G. max* (Gm), and *M. truncatula* (Mt) were sequenced using ClustalW [56] to determine the relationships of the MTP family with other plants. The phylogenetic tree was structured via the MEGA 7.0 software [57] using the neighbor-joining method (1000 bootstrap replications). All identified *PvMTP* genes’ chromosome positions were obtained from the Phytozome 13 (https://phytozome-next.jgi.doe.gov/phytomine/begin.do/ accessed on 4 December 2022) online database. The locations of the *PvMTP* genes on the chromosomes were marked using the PhenoGram Plot (https://visualization.ritchielab.org/ accessed on 4 December 2022).

### 3.4. Identification of Exon–Intron Structures and Protein Motifs

Whole genome and CDS sequences of *A. thaliana* (At) and *P. vulgaris* (Pv) MTP genes were obtained from the Phytozome 13 (https://phytozome-next.jgi.doe.gov/phytomine/begin.do / accessed on 4 December 2022) online database. The exon–intron regions of the obtained data were defined using Gene Structure Display Server V2.0 (http://gsds.gao-lab.org/ accessed on 4 December 2022) [58]. Chromosome size (bp), chromosomal location, and intron numbers were determined using the Phytozome database. Conserved motifs in MTP sequences were retrieved from the online database MEME (https://meme-suite.org/meme/tools/meme/ accessed on 5 December 2022) tool with the following parameters: a maximum number of motifs of 10 and a min/max motif width of 6–50 [59]. The tBtools software program was used for the visual enhancement of the motifs in bean MTP genes [60] and identified conserved motif sequences that were verified via InterPro 91.0 (https://www.ebi.ac.uk/interpro/result/InterProScan/ accessed on 4 December 2022) and HMMER 2.41.2 (https://www.ebi.ac.uk/Tools/hmmer/search/phmmer/ accessed on 4 December 2022). Arabidopsis and common bean MTP sequences were aligned using the MEGA-11 (https://www.megasoftware.net/ accessed on 5 December 2022) tool, and motif regions were visualized using the GeneDoc 2.7 software [61,62].

### 3.5. Promoter Region Cis Motif Analysis, Subcellular Localization, and Protein–Protein Interactions

For the cis-regulatory element analysis, the promoter region sequence information of the *PvMTP* protein sequences was utilized. All gene sequence information was obtained from the Phytozome 13 online database, and a 1500 bp area was detected from the transcription start site [39]. Cis-regulatory element analysis was performed using the online PlantCARE database (https://bioinformatics.psb.ugent.be/webtools/plantcare/html/ accessed on 6 December 2022) [27,39]. The MTP protein sequences were used to predict the localization of the gene in the cell and determined via the online database CELLO v.2.5:18ubcellularr Localization predictor (http://cello.life.nctu.edu.tw/ accessed on 6 December 2022). The proportions of the determined regions were obtained via the WoLF PSORT (https://wolfpsort.hgc.jp accessed on 6 December 2022) database. Whether the MTP proteins had a nuclear localization signal was checked via Rostlab (https://rostlab.org/services/nlsdb/accessed on 6 December 2022). A heatmap was generated for the predicted localized genes via TBTool. The MTP protein sequences were analyzed using the STRING 11.5 tool (https://string-db.org/ accessed on 7 December 2022) to identify protein–protein interactions.

### 3.6. Primer Design and qRT PCR Analysis

The protein sequences of the metal tolerance genes obtained after the similarity analyses between *A. thaliana* and *P. vulgaris* were taken from the Phytozome v13 databases. The primers to be used were designed via Primer3 v.4.1.0 (https://primer3.ut.ee/ accessed on 1 December 2022) [63,64]. The primers were planned to be Tm 60 ± 1 °C and to have a PCR amplicon size of 73–151 bp, a primer sequence length of 20–22 nucleotides, and a GC ratio of 45–65%. The *PvMTP4*, *PvMTP5*, and *PvMTP12* primers are presented in Table 4. During the primer design, exon hybrid regions were selected to reduce genomic DNA contamination. The sequence information of the housekeeping gene Beta Actin was obtained from the study by Yeken et al. [65].

*PvMTP*s expression was analyzed using the SYBR^®^ Green Supermix (Bio-Rad, Hercules, CA, USA). The following reaction stages were used in the amplifications on the Real-Time PCR (CFX-Connect Bio-Rad, Hercules, CA, USA) apparatus: beginning denaturation at 50 °C for 60 min, followed by conditions of 95 °C for 5 min. At this point, the fluorescence signal was recorded during 40 cycles for each step at 95 °C for 10 min, 60 °C for 30 s, and 72 °C for 5 s. A final stage at 95 °C for 10 s with fluorescence readings at each 0.5 °C fluctuation (from 65 to 95 °C) was added to create the melting curve. There were three biological replicas of each reaction, which were each run in duplicate.

### 3.7. RNA Extraction, DNAse Treatment, and cDNA Synthesis

The total RNA (100 mg) from all sampled tissues was isolated using the Wiz-Prep plant RNA isolation kit according to the manufacturer’s protocol (https://www.wizbiosolution.com/ accessed on 15 September 2022). Total RNA was isolated from genomic DNA using dNase I, rNase-free (Thermo: EN0525). A nano spectrophotometer (Denovix, Wilmington, DE, USA) was used to assess the quantity and quality of the RNA. The iScriptTM cDNA synthesis kit (Bio-Rad, Hercules, CA, USA) was used to create complementary DNA after following the manufacturer’s instructions. For additional research, each sample was properly frozen at 80 °C.

### 3.8. Plant Growth, Fe and Zn Applications

The BN-16, SV-12, EL-29, and MS-46 genotypes were used in this study. The water culture method used to cultivate the plants was modified from de-Figueiredo et al. [66]. Seeds were sterilized with a 10% sodium hypochlorite (NaClO) solution for 1 min, rinsed with distilled water, and germinated in perlite at 22 °C for 5–7 days. Young seedlings were transferred to hydroponic pots, including nutrient solutions prepared according to Hoagland and Arnon [67]. The plants were grown in the climate chamber at 24 °C with 70% humidity under a 16-h light/8-h dark cycle. One plant was grown in each pot, and five plants were used as replicas. The experiment was performed in three replications. To the BN-16 and SV-12 genotypes were applied zinc doses of 0 (control), 10 µM of ZnCl_2,_ and 15 µM of ZnCl_2_. To the EL-29 and MS-46 genotypes were applied iron doses of 0 (control) and 15 and 30 µM of Fe (III)-Eddha. The Fe (III)-Eddha and ZnCl_2_ applications were started at the beginning of the flowering of the plants. The applications were conducted once a week on the same day and at the same time. During the application, the nutrient solutions were changed. The pH level of the nutrient solution was checked daily. After pollination and fertilization, the pods were harvested at different developmental stages (^3r^d, ^6t^h, 1^2t^h, and 2^4t^h days). Root harvesting was done after the 2^4t^h day of sampling. After harvest, the roots and pods were rapidly frozen in liquid nitrogen for RNA isolation; then, they were kept at −80 °C until analysis. The samples taken for Fe and Zn analysis in tissues were dried in an oven at 70 °C. Then, 1 gram of the ground samples was weighed, and wet digestion was performed according to the method of Kacar [68]. The readings of the samples were taken at the Van Yüzüncü Yıl University Central Research Laboratory using an atomic absorption spectrophotometer (Thermo Scientific/ICE-3000 series, Thermo, Cambridge, United Kingdom).

### 3.9. Data Analyses

The expression profiles of the *PvMTP4*, *PvMTP5*, and *PvMTP12* genes were calculated via the 2^−ΔΔCT^ method, and all data were log_2_ (treated expression level/control expression level) transformed to enhance normality [69]. The study examined the effects of genotype, dose, and time factors. Analysis of variance (3-way ANOVA) was used to determine the singular and interactive effects of factors. Student’s t (LSD) multiple comparison test was used to compare the mean doses of zinc and iron (α = 0.05). Statistical analyses were conducted using the R Studio program [70].

## 4. Conclusions

In this study, the metal tolerance protein (MTP) gene family of the common bean was identified via genome-wide analysis that focused on the effect of Fe and Zn treatments on the *PvMTP* genes. The gene expression levels of three genes in the Zn-CDF (*PvMTP*4,5,12) subgroup were analyzed after Fe and Zn treatments. Four common bean genotypes with different Fe and Zn storage abilities were used to elucidate the responses of the genes. The highest expression levels of the *PvMTP*4,5,12 genes were observed after the Zn treatments. The amount of Zn measured in the pods and roots of the SV-12 genotype was confirmed via the gene expression results. The differences found in the Fe and Zn contents stored in tissues confirm that there are significant differences within bean gene sources. In addition to providing a basis for the physiological characterization of common bean MTPs, this study demonstrates the potential of MTPs in the biofortification of legumes with iron and zinc.

## Figures and Tables

**Table 1:** Characteristics of *PvMTP* genes in *Phaseolus vulgaris*.

Gen ID	Phytozome ID	Chr: Location	Protein				
			Domain Family	Length (aa)	kDa	pI	Instability Index
*PvMTP4*	Phvul.011G061200.1	Chr11: 5461254..5462423	PF01545	389	42.47	5.94	25.85
*PvMTP5*	Phvul.001G142750.1	Chr1: 38584559..38590051	PF01545	369	41.35	7.85	37.63
*PvMTP6*	Phvul.007G165166.1	Chr7: 27868992..27875439	PF01545-PF16916	490	53.21	6.55	45.77
*PvMTP7*	Phvul.008G254900.1	Chr8: 60275955..60280336	PF01545	422	46.77	8.97	35.47
*PvMTP8*	Phvul.006G080700.1	Chr6: 19280258..19283725	PF01545-PF16916	413	46.16	5.44	50.45
*PvMTP9*	Phvul.011G178800.1	Chr11: 48990796..48998055	PF01545-PF16916	407	46.47	6.77	46.31
*PvMTP10*	Phvul.005G049300.1	Chr5: 5742399..5745782	PF01545-PF16916	396	45.42	8.36	41.51
*PvMTP11*	Phvul.008G015500.1	Chr8: 1278329..1281961	PF01545-PF16916	396	44.98	5.08	39.58
*PvMTP12*	Phvul.002G176300.1	Chr2: 33458940..33461381	PF01545	813	91.05	6.67	45.33

**Table 2:** Motif information of protein sequences of *PvMTP*s.

Motif	Motif Sequence
**Motif 1**	TLDSLLDLLSGFILWFTAYAMRNPNQYQYPIGKKRMQPLGIIVFASVMAT
**Motif 2**	DTVRAYTFGAHYFVEVDIVLPEDMPLQZAHNIGESLQEKLEQLPEVERAF
**Motif 3**	MIYCRRFKNEIVRAYAQDHFFDVITNVVGLVAAVLAVKYYWWIDPIGAII
**Motif 4**	ALYTINTWARTVIENVWSLIGRSAPPDFLQKLTYLIWNHHE
**Motif 5**	RKVAEYYKQQZKLLEGFTEMDSJTETGYFPGALSEDEMKQ
**Motif 6**	SNAANJVLFVAKVYASIRSGSLAIIAS
**Motif 7**	VHJDFEFTHKPEH
**Motif 8**	KPNMDPEQLQWMIGIMLSVTIVKFIL
**Motif 9**	SDAAHLLFDCAALSISLFAVWASRWPABRHYSYGYGRLEVLSAFTN

**Table 3:** Frequency and function of cis-regulatory elements in putative promoter regions of the 1500 bp upstream region of *PvMTP* genes (yellow: cis-acting element; green: stress and growth elements; pink: hormone-sensitive elements).

Motif	Putative Function	*PvMTP*4	*PvMTP*5	*PvMTP*6	*PvMTP*7	*PvMTP*8	*PvMTP*9	*PvMTP*10	*PvMTP*11	*PvMTP*12
**TATA-box**	core promoter element	15	25	21	14	36	71	10	12	7
**Box III**	protein binding site	0	0	0	0	0	0	1	0	0
**CCAAT-box**	MYBHv1 binding site	0	1	0	1	0	0	1	0	1
**CAAT-box**	promoter and enhancer regions	12	16	8	13	6	6	4	7	4
**AT-rich element**	AT-rich (ATBP-1)	1	1	1	0	0	0	1	0	0
**HD-Zip 3**	protein binding site	0	0	0	0	0	0	1	0	0
**G-box**	light responsiveness	0	0	1	3	0	1	1	0	0
**GT1-motif**	light responsiveness	0	0	0	1	2	1	2	0	3
**3-AF1 binding site**	light responsiveness	0	0	0	0	1	0	1	0	0
**TCCC-motif**	light responsiveness	0	0	1	0	0	0	1	0	6
**GATA-motif**	light responsiveness	0	1	0	0	0	0	0	0	1
**Sp1**	light responsiveness	0	0	0	1	0	0	0	0	2
**AE-box**	light responsiveness	0	2	0	0	0	1	0	0	1
**I-box**	light responsiveness	1	1	0	0	0	0	0	0	1
**LAMP-element**	light responsiveness	1	0	0	0	0	0	0	0	0
**MRE**	light responsiveness	2	1	0	0	0	0	0	0	0
**TCT-motif**	light responsiveness	0	2	0	2	0	1	0	0	0
**chs-CMA2a**	light responsiveness	0	1	0	0	0	0	0	0	0
**Box 4**	light responsiveness	0	2	0	1	0	7	0	0	0
**ATCT-motif**	light responsiveness	0	0	1	0	0	0	0	0	0
**chs-CMA1a**	light responsiveness	0	0	1	0	0	1	0	0	0
**ATC-motif**	light responsiveness	0	0	0	0	1	0	0	0	0
**AT1-motif**	light responsiveness	0	0	0	0	0	4	0	0	0
**LTR**	low-temperature responsiveness	1	1	1	1	0	0	0	0	2
**MBS**	drought inducibility	0	1	0	1	1	1	1	1	0
**O2-site**	zein metabolism regulation	1	0	1	0	0	0	0	1	0
**circadian**	circadian control	0	0	0	0	1	0	0	1	0
**ARE**	anaerobic induction	4	0	3	3	0	2	8	2	1
**CAT-box**	meristem expression	0	0	1	3	0	0	1	0	0
**TC-rich repeats**	defense and stress responsiveness	1	1	0	0	0	1	1	2	1
**TGA-element**	auxin-responsiveness	0	0	0	2	0	0	1	1	0
**AuxRR-core**	auxin responsiveness	0	0	0	0	0	0	0	1	0
**CGTCA-motif**	MeJA-responsiveness	0	2	4	1	0	0	3	0	1
**G-Box**	MeJA-responsiveness	0	2	0	0	0	0	1	0	0
**TGACG-motif**	MeJA-responsiveness	0	2	4	1	0	0	3	0	1
**ABRE**	abscisic acid responsiveness	0	2	2	2	0	0	4	0	0
**TCA-element**	salicylic acid responsiveness	0	0	1	0	0	2	1	0	0
**GARE-motif**	gibberellin-responsiveness	1	0	0	0	1	1	0	1	0

**Table 4:** Primer information in *Phaseolus vulgaris* L.

Primer	Phytozome ID	Size	Sequence Information
***PvMTP*4 F.** ***PvMTP*4 R.**	Phvul.011G061200.1	73 bp	5′-AATCTTCAGGGGGCGTATTTGC-3′ 5′-TGGCTCCAGCAATCATCACTCC-3′
***PvMTP*5 F.** ***PvMTP*5 R.**	Phvul.001G142750.1	151 bp	5′- AGGGCAACTGGTGGTATCTTG-3′ 5′-CGATCACATGACCTGGCACTA-3′
***PvMTP*12 F.** ***PvMTP*12 R.**	Phvul.002G176300.1	113 bp	5′-TGATCCCGCCTGCTCAATTT-3′ 5′-CATGTTCTTGCACCCTTGGC-3′
**Beta Aktin F.** **Beta Aktin R.**	-	-	5′-TGAGCAAGGAGATTACAGCATTGG-3′ 5′- CATACTCTGCCTTCGCAATCCAC-3′

## Data Availability

All data contained within the article.

## Supplementary Materials

The following supporting information can be downloaded at: https://www.mdpi.com/article/10.3390/plants12183218/s1, Figure S1: Expression levels of *PvMTP* (4, 5, 12) genes in the pod of common beans genotypes under Fe (0, 15 and 30 µM) and Zn (0, 10, 15 µM) application in harvested a different day (Different letters indicate significant differences according to Student’s t-test, * (*p* ≤ 0.05); ** (*p* ≤ 0.01); *** (*p* ≤ 0.001); ns: non-significant).; Figure S2: Fe and Zn amount in pod (A) and root (B) of common beans genotypes under Fe (0, 15 and 30 µM) and Zn (0, 10, 15 µM) applications (Different letters indicate significant differences according to Student’s t-test, * (p ≤ 0.05); ** (p ≤ 0.01); *** (p ≤ 0.001); ns: non-significant).
