# Supplementary material for: Genome-Wide Identification and Expression Analysis of MTP (Metal Ion Transport Proteins) Genes in the Common Bean
Source: Plants (Basel). 2023 Sep 9;12(18):3218. doi: 10.3390/plants12183218 (PMC10535811; doi:10.3390/plants12183218)
Supplement: Supplementary file 1 [file plants-12-03218-s001.zip › plants-2584908-supplementary.pdf]

Supplementary Material

Genome-Wide Identification and Expression Analysis of MTP (Metal Ion Transport Proteins) Genes in the Common Bean

Hilal Yilmaz [1,2], Göksel Özer [3], Faheem Shehzad Baloch [4,*], Vahdettin Çiftçi [2], Yong Suk Chung [5] and Hyeon-Jin Sun [6,*]

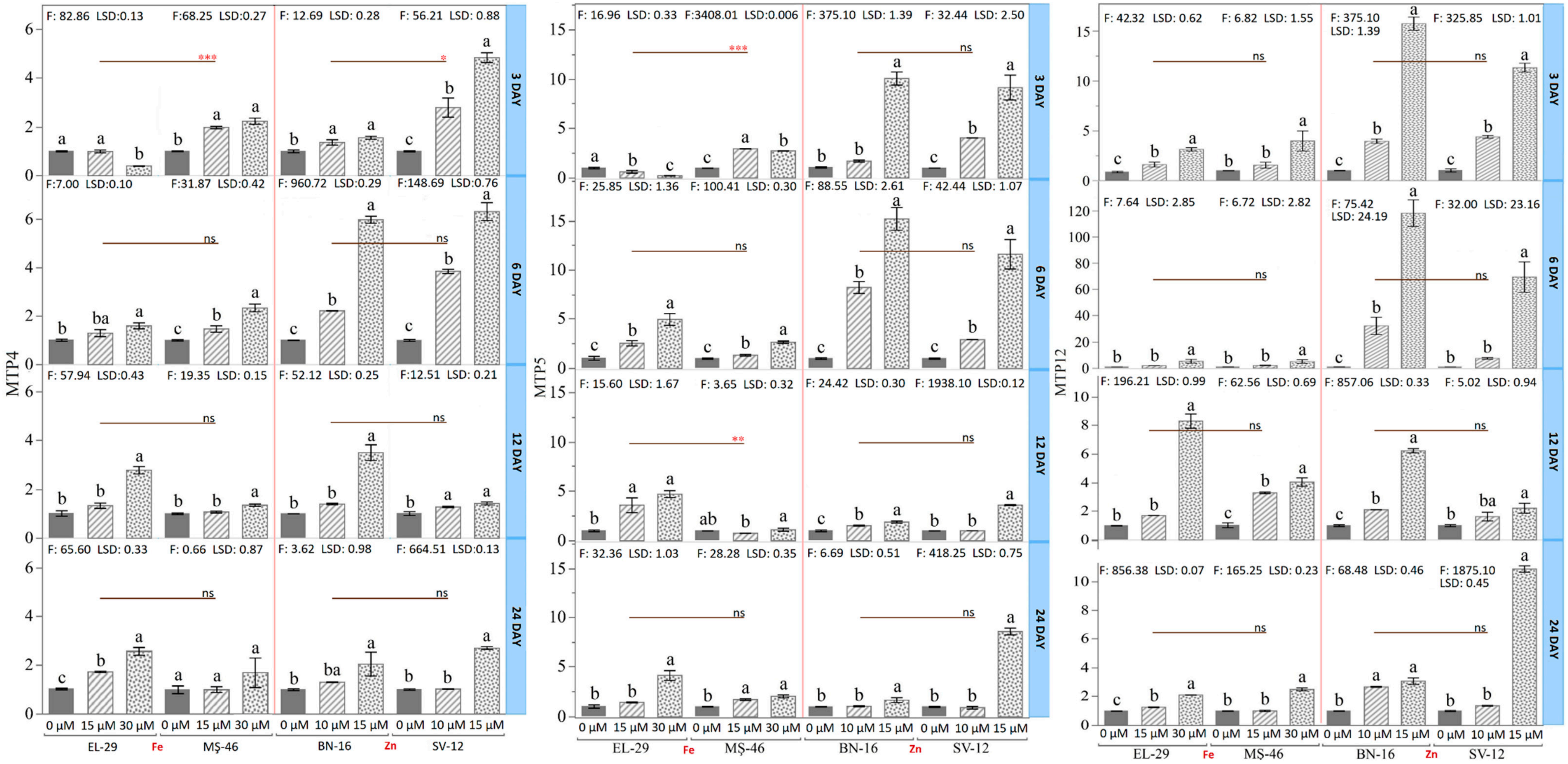

Figure S1. Expression levels of *PvMTP* (4, 5, 12) genes in the pod of common beans genotypes under Fe (0, 15 and 30 µM) and Zn (0, 10, 15 µM) application in harvested a different day (Different letters indicate significant differences according to Student's t-test, * ($p \leq 0.05$); ** ($p \leq 0.01$); *** ($p \leq 0.001$); ns: non-significant).

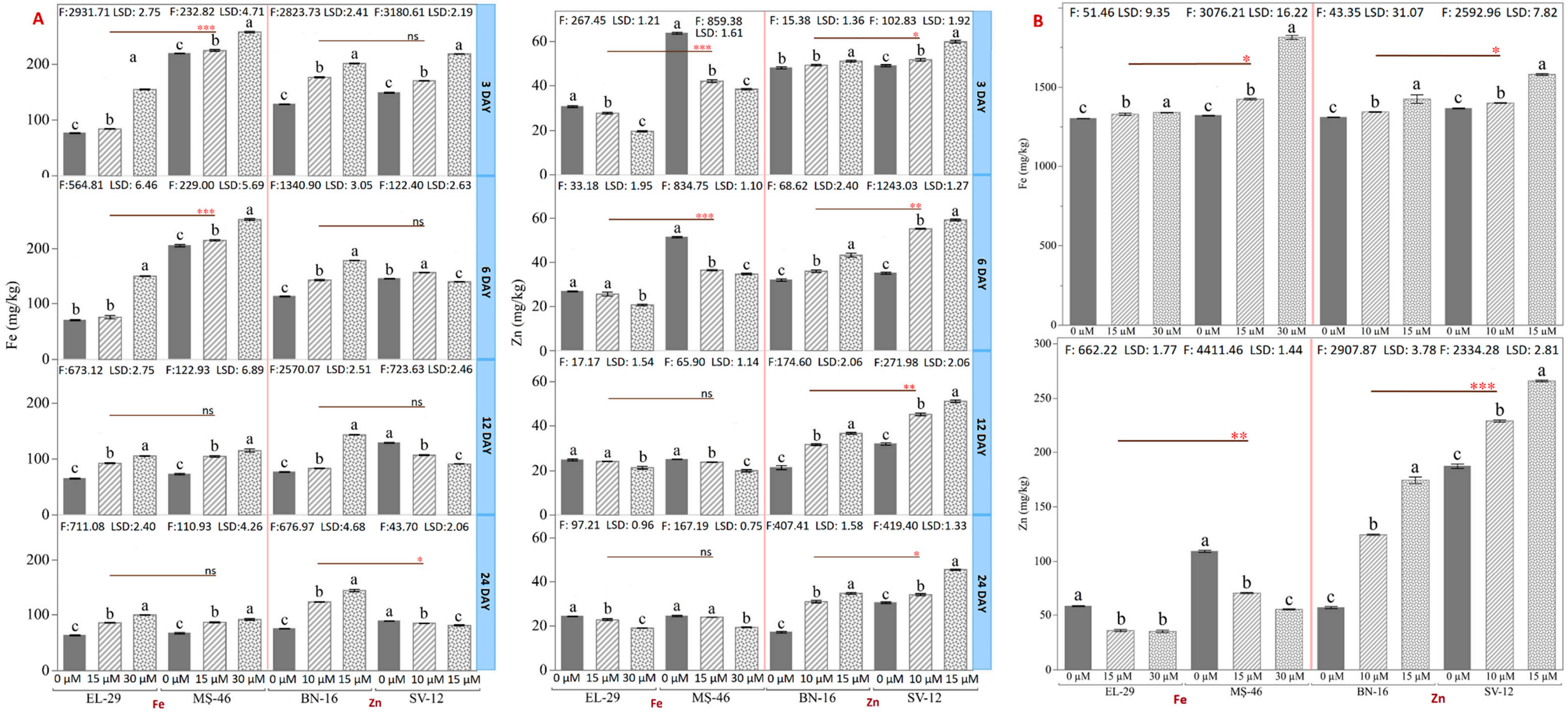

Figure S2. Fe and Zn amount in pod (**A**) and root (**B**) of common beans genotypes under Fe (0, 15 and 30 μM) and Zn (0, 10, 15 μM) applications (Different letters indicate significant differences according to Student's t-test, * ($p \leq 0.05$); ** ($p \leq 0.01$); *** ($p \leq 0.001$); ns: non-significant).
